# Outcome and Challenges in the Treatment of Pediatric Hodgkin Lymphoma With Euronet‐PHL‐C1 Protocol: Experience From a Resource‐Limited Country

**DOI:** 10.1002/cam4.71095

**Published:** 2025-07-31

**Authors:** Elie Bechara, Toufic Eid, Arwa El‐Dhuwaib, Hani Tamim, Dolly Noun, Raphah Borghol, Zaher Chakhachiro, Miguel R. Abboud, Samar Muwakkit

**Affiliations:** ^1^ Department of Pediatrics and Adolescent Medicine, Faculty of Medicine American University of Beirut Beirut Lebanon; ^2^ Children's Cancer Institute American University of Beirut Medical Center Beirut Lebanon; ^3^ Department of Radiation Oncology American University of Beirut Medical Center Beirut Lebanon; ^4^ Department of Internal Medicine Clinical Research Institute, American University of Beirut Medical Centre Beirut Lebanon; ^5^ College of Medicine, Alfaisal University Riyadh Saudi Arabia; ^6^ Department of Pathology and Laboratory Medicine American University of Beirut Medical Center Beirut Lebanon

**Keywords:** children, euronet protocol, Hodgkin lymphoma, low‐ and middle‐income country (LMIC), pediatric, radiotherapy

## Abstract

**Background:**

The Euronet‐PHL‐C1 protocol has yielded excellent results for pediatric Hodgkin Lymphoma (HL), by omitting radiotherapy (RT) in early responders, thereby decreasing long‐term toxicities. However, its application in resource‐limited countries remains challenging. This study aims to evaluate patient outcomes using this protocol and the feasibility of omitting RT for early responders.

**Methods:**

We conducted a retrospective analysis of 87 previously untreated pediatric HL patients at our Center from 2012 to 2022, following the Euronet‐PHL‐C1 protocol. RT was omitted for patients with an early rapid response at interim evaluation. Collected data were analyzed to determine survival outcomes and predictors of relapse.

**Results:**

The mean age of the patients was 13 years, with 51.7% female. B‐symptoms were present in 59.8% of the patients, while 37.9% had bulky disease, 57.1% had elevated erythrocyte sedimentation rate, and 42.5% had stage IV disease. RT was omitted for early rapid responders in 22.9% of the patients. The therapy was generally well tolerated, with only 36 episodes of febrile neutropenia and no treatment‐related mortality. The 5‐year progression‐free survival and overall survival of the entire cohort were 89.3% and 97.6%, respectively. Nine patients relapsed, and two patients died. No independent predictors of event‐free survival were identified.

**Conclusion:**

The implementation of Euronet‐HL protocol in our center provided excellent outcomes and a safety profile despite a few challenges. While RT can be removed in low‐stage, rapid‐responder patients, caution persists in resource‐limited settings for those with advanced stage or bulky disease, highlighting the need for prospective trials to guide safe RT omission.

**Abbreviations**
ABVD: adriamycin, bleomycin, vinblastine, dacarbazine
COPDAC: cyclophosphamide, vincristine, prednisone, dacarbazine
CT: computed tomography
EBV: Epstein–Barr virus
ESR: erythrocyte sedimentation rate
FN: febrile neutropenia
G‐CSF: granulocyte colony stimulating factor
Gy: gray
HIC: high‐income countries
HL: Hodgkin lymphoma
LMIC: low‐ and middle‐income countries
MENA: middle East and North Africa
OEPA: vincristine, etoposide, prednisone, adriamycin
OS: overall survival
PET‐CT: positron emission tomography‐computed tomography
PFS: progression‐free survival
RT: radiotherapy
TG: treatment group

## 1 Introduction

Hodgkin Lymphoma (HL) is one of the most common cancers in adolescents and the third most common malignancy in children [1]. Over recent decades, treatment outcomes for pediatric and adolescent patients with HL have improved significantly, with overall survival rates now approaching 98% [2, 3]. This achievement is largely due to advancements in diagnostic tools, which have enhanced precision in detecting active disease sites and assessing treatment response, as well as improvements in disease management. However, this success has come at the cost of long‐term toxicities from chemotherapy and radiotherapy (RT) in survivors. As overall survival is no longer a primary concern and current treatment protocols yield excellent outcomes, the focus has shifted toward minimizing long‐term morbidity. This involves modifying existing protocols by omitting agents associated with significant toxicities and identifying patient subgroups in whom radiation can be safely avoided. For patients requiring radiation, efforts are concentrated on safely reducing the irradiated area and carefully lowering the radiation dose [4].

Nowadays, the majority of pediatric oncology groups are abandoning adult protocols of HL based on the ABVD regimens (Adriamycin, Bleomycin, Vinblastine, Dacarbazine) and systematic RT, which were responsible for cardiotoxicities, pulmonary fibrosis, infertility, and secondary malignancies in long‐term survivors [5]. The German Society of Pediatric Oncology and Hematology (GPOH) was the first to investigate the use of a risk‐adapted and response‐based approach, which resulted in excellent outcomes and allowed the omission of RT in low‐risk patients who showed early complete remission [6]. These excellent results encouraged the European pediatric and adolescent Hodgkin lymphoma network (Euronet‐HD) to develop the Euronet‐PHL‐C1 regimen (NCT00433459) in 2007 to treat children with classic Hodgkin Lymphoma [7, 8]. This protocol consisted of using risk‐adapted management based on the stage of the disease and response assessment using positron emission tomography‐computed tomography (PET‐CT). For low‐risk patients, the OEPA regimen (Vincristine, Etoposide, Prednisone, Adriamycin) was used; as for other stages, the OEPA and COPDAC (cyclophosphamide, Vincristine, Prednisone, Dacarbazine) regimens were given. Using this approach, there were lower cumulative doses of anthracyclines and alkylating agents. The risk of pulmonary fibrosis with bleomycin was removed, and gonadal toxicity was decreased by replacing Procarbazine with Dacarbazine [9, 10]. RT was administered only to patients who did not achieve adequate early response.

Publications on pediatric HL from the Middle East and North Africa (MENA) region are scarce in general, with the majority of chemotherapeutic protocols used being the ABVD regimen [11, 12, 13, 14, 15, 16, 17, 18] mainly because of the influence of adult practice. Other causes include drug availability, cost of treatment, limitations in incorporating PET‐CT for initial staging and response assessment, and finally, the lack of awareness of long‐term toxicities [19]. Other studies have used a hybrid chemotherapy regimen: ABVD for low‐risk and OEPA/COPDAC for high‐risk patients [18, 20, 21]. Only two studies from LMIC have published their outcomes using the Euronet‐HL protocol for all risk groups; one from India [19] and another from Pakistan [22]. The results were encouraging, but there were concerns regarding treatment abandonment and treatment‐related mortality.

Until 2012, children with HL were being treated at our center according to CCG 5942 (COPP/ABV) [23], with IFRT being administered to patients who did not achieve a complete response to initial chemotherapy or at the discretion of the treating physician [24]. This strategy resulted in an inferior survival outcome for advanced‐stage patients treated with chemotherapy alone, making RT warranted [25]. Moreover, this approach led to an increased number of patients receiving RT and a rise in infertility cases among boys (unpublished data). In order to enhance survival while reducing long‐term toxicities, exploring new therapeutic strategies was crucial. Therefore, following consultation with St. Jude Children's Research Hospital—given our institutional affiliation—we chose to implement the Euronet protocol over the COG protocol, aiming to eliminate bleomycin and procarbazine and reduce exposure to anthracyclines and radiation therapy. Our primary aim in this retrospective study was to evaluate the outcome as well as the adverse events of our patients treated with the Euronet‐PHL‐C1 protocol. The secondary aim was to report the percentage of patients who were able to omit RT from their treatment.

## 2 Methods

### 2.1 Patients

This was a retrospective chart review study of patients younger than 18 years, with a histologically confirmed HL, treated according to the Euronet‐PHL‐C1 protocol at the Children Cancer Institute (CCI) of the American University of Beirut Medical Center (AUBMC) in Lebanon, between January 2012 and December 2022. Collected data included patients and disease characteristics, information about treatment, toxicity, outcome, and survival. Patients were followed for the occurrence of any event.

### 2.2 Staging

All children underwent CT scan and PET‐CT at diagnosis, early response evaluation (after 2 OEPA), and at the end of treatment. Patients were classified into clinical stage according to the classification of Ann‐Arbor [26] and they were assigned to treatment group (TG) as defined by the protocol (Table 1). The majority of patients had bilateral bone marrow biopsy for staging. Erythrocyte sedimentation rate (ESR) was considered elevated if more than 30 mm/h. Bulky disease was defined as a single or conglomerate node volume of more than 200 mL. After the amendment made by the Euronet group in 2016, patients originally assigned to TG1 and having either elevated ESR or bulky disease were upstaged to TG2. Twenty‐seven patients (31%) were classified according to the old criteria.

**TABLE 1:** Treatment groups based on staging as per Euronet‐PHL‐C1 protocol.

Treatment group (TG)	Stage
TG1	I A/B, II A
TG2	I EA/B, II EA, II B, III A I A/B, II A with ESR > 30 mm or Bulky disease (> 200 mL)^a^
TG3	II EB, III EA, III B, IV A/B

^a^ After the amendment made by the Euronet group in 2016, patients originally assigned to TG1 and who have either elevated ESR or bulky disease were upstaged to TG2.

### 2.3 Treatment

All patients received 2 cycles of OEPA, with granulocyte colony stimulating factors (G‐CSF) given after each cycle for at least 5 days and continued until absolute neutrophil count was higher than 500 cells per mm^3^. Additional 1, 2, or 4 cycles of COPDAC were administered to TG1, TG2, and TG3 groups, respectively. RT was administered at a total dose of 19.8 Gy in 11 fractions to all initially involved sites but only for patients who showed an inadequate response after the interim evaluation following two cycles of OEPA. An inadequate response corresponded to less than 50% tumor volume reduction in any involved nodal site on CT scan or a visual Deauville score of 3 or higher on PET‐CT. An additional boost of 10 Gy was administered to patients who had less than 75% response and still had more than 5 mL residual volume or residual disease more than 100 mL. The field of radiation used for treatment is modified involved field, defined as pre‐chemotherapy nodal volume with a 1–2 cm margin, except in the mediastinum and para‐aortic and pelvic areas, where post‐chemotherapy volume is contoured instead. Patients were followed up three monthly in the first 2 years, four monthly in the third year, and six monthly in the fourth and fifth year after achieving remission. Yearly evaluations included: echocardiography and electrocardiogram, thyroid function studies, and ultrasound.

### 2.4 Statistical Analysis

Statistical analysis was performed using SPSS statistical package version 25.0 (SPSS Inc., Chicago, IL). Continuous data were described as mean and standard deviation (sd), and median and interquartile range (IQR). Frequencies and percentages were used to describe categorical variables. Survival distributions were examined using Kaplan–Meier curves; event‐free survival (EFS) was defined as time from treatment start until the first of the following events: death from any cause, disease progression, relapse, or last follow‐up. Overall survival (OS) was defined as time from start of treatment to last follow‐up or death. Univariate and multivariate hazard ratios were analyzed by Cox proportional hazards regression, where results are reported as hazard ratio (HR) and 95% confidence interval (CI). Statistical significance was set at *p*‐value < 0.05.

### 2.5 Ethical Considerations

Ethical approval was obtained from the Institutional Review Board of the American University of Beirut (IRB: BIO‐2022‐0051) for chart review and data collection. Consent from parents/guardians was not required, as the study was retrospective.

## 3 Results

### 3.1 Epidemiological and Clinical Data

During the eleven‐year period, the charts of 128 patients were reviewed, but only 87 patients with HL were included in the study (14 patients partially treated at our center, 10 patients did not have HL, and 17 patients were radiated by physician preference against protocol). Demographic and clinical data are summarized in Table 2. Seventy‐nine patients (90.8%) were Lebanese; the rest were from nearby countries. The mean age at diagnosis was 13.1 years (range 2–17 years), with 51.7% being female. The mean duration of symptoms before diagnosis was 3.2 months (range: 1 week‐1 year). The most common presenting sign was painless neck mass in 52 patients (59.8%). B‐symptoms were present in 52 patients (59.8%), while 33 patients (37.9%) had bulky disease, 48 patients (57.1%) had elevated ESR, and 37 patients (42.5%) had stage IV disease.

**TABLE 2:** Baseline demographic and clinical characteristics of the cohort (*N* = 87).

Variables	*N* (%)
Age	
Mean ± SD	13.1 ± 3.8
Median (IQR)	15.0 (10.0–16.0)
≥ 13 years	56 (64.4%)
Female	45 (51.7%)
Positive Family history of malignancy	37 (42.5%)
Symptoms' duration (weeks)	
Mean ± SD	13.0 ± 13.0
Median (IQR)	8.0 (4.0–18.0)
B‐symptoms	52 (59.8%)
ESR (mm)	
Mean ± SD	46.4 ± 36.4
Median (IQR)	40.00 (14.50–66.00)
ESR ≥ 30 mm	48 (57.1%)
EBV status	
Immune	51 (58.6%)
Not immune	17 (19.5%)
Active disease	3 (3.4%)
Not Available	16 (18.4%)
Organ involvement	
Lung	21 (24.1%)
Spleen	29 (33.3%)
Liver	4 (4.6%)
Bone	17 (19.5%)
Bone marrow	9 (10.3%)
Bulky disease	33 (37.9%)
Pathology	
Nodular sclerosis	61 (70.1%)
Mixed cellularity	6 (6.9%)
Lymphocyte depleted	1 (1.1%)
Not specified	19 (21.8%)
Positive interim PET	26 (30.2%)
Staging	
Stage I	1 (1.2%)
Stage II	29 (33.3%)
Stage III	20 (23.0%)
Stage IV	37 (42.5%)
Time to start chemotherapy (days)	
Mean ± SD	11.2 ± 6.5
Median (IQR)	10.0 (6.0–14.0)
Radiotherapy	61 (70.1%)
B‐symptoms & Bulky disease	20
B‐symptoms only	20
Bulky disease only	5

Abbreviations: EBV, Epstein–Barr virus; ESR, erythrocyte sedimentation rate; IQR, interquartile range; PET, positron emission tomography; SD, standard deviation.

### 3.2 Treatment

**FIGURE 1:**
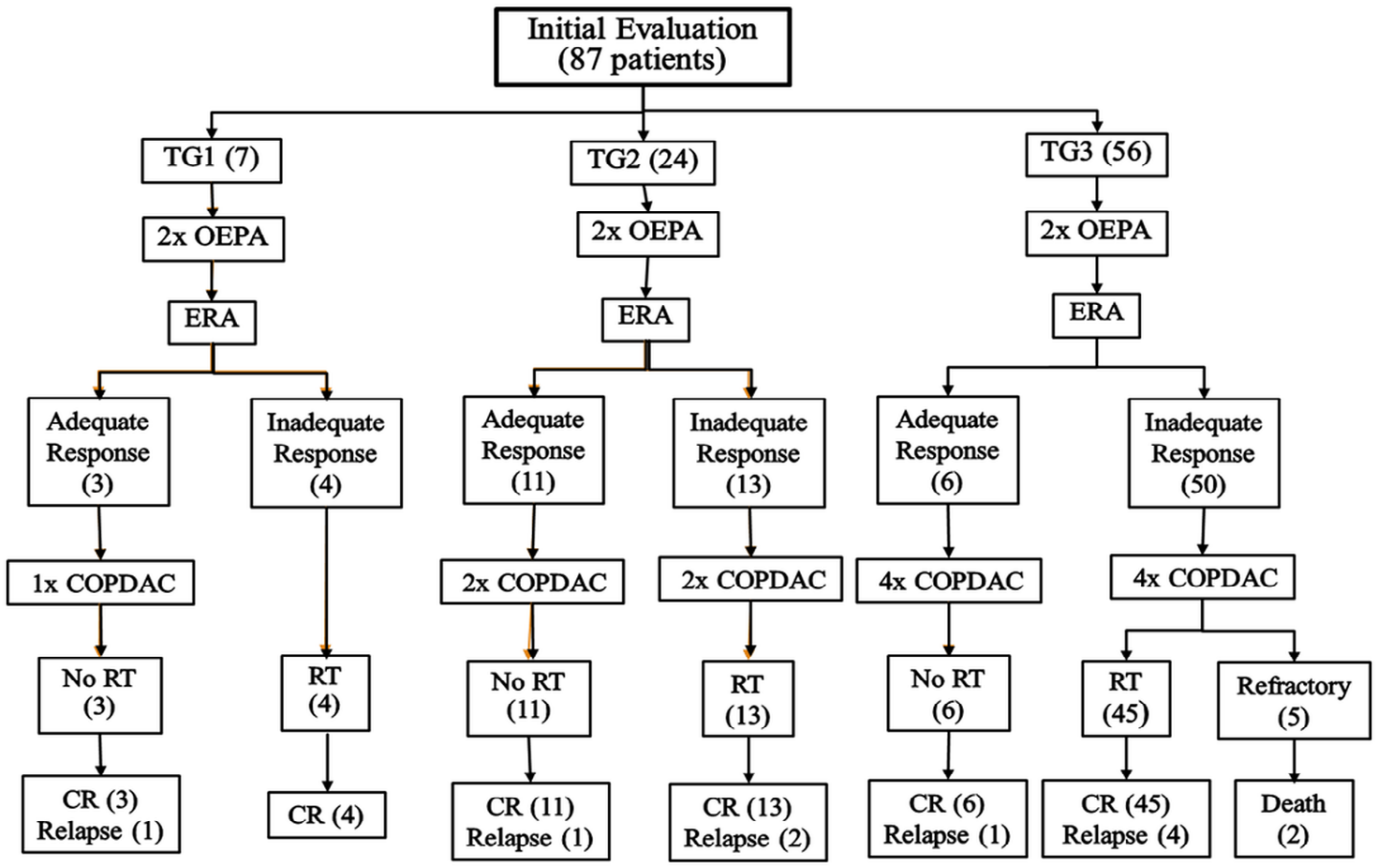
Treatment algorithm and outcome for pediatric Hodgkin Lymphoma based on Euronet‐PHL‐C1 protocol. COPDAC, cyclophosphamide, oncovin, prednisone, dacarbazine; CR, complete remission; ERA, early response assessment; OEPA, oncovin, etoposide, prednisone, adriamycine; RT, radiotherapy; TG, treatment group.

Treatment administration and outcome are illustrated in Figure 1. All patients received chemotherapy according to the protocol. There was no treatment abandonment. There were 7 patients (8.1%) treated with TG1 (with 3 patients receiving an additional COPDAC cycle as per Euronet amendment after 2016), 24 patients (27.6%) treated with TG2, and 56 patients (64.3%) received TG3. Early response evaluation was available for all patients and showed inadequate response in 61 out of the 87 evaluable patients (70.1%), and received RT according to the protocol. Out of the 26 patients who did not receive radiotherapy, 20 patients (22.9%) had adequate response as per protocol, 5 patients (5.7%) were refractory to first‐line therapy, and one patient (1.1%) had extensive disease and radiotherapy deferred because of expected excessive toxicity.

### 3.3 Toxicity

Therapy was generally well tolerated; there was no treatment‐related mortality. Most adverse events occurred as Febrile Neutropenia (FN) with 36 episodes in the 87 evaluable patients, with the majority (83.3%) developing during the 2 cycles of OEPA. Although incomplete, the list of other acute side effects included severe infections (either bacterial, fungal, or viral infections) in 23 patients (26.4%), osteonecrosis in 13 patients (14.9%), sensory or motor neuropathy in 22 patients (25.3%), central line complications in 9 patients (10.3%), and cardiac toxicity requiring medication in 3 patients (3.4%). Radiotherapy‐related toxicity included grade II skin and/or gastrointestinal irritation in 8 patients (9.1%). Two patients developed macrophage‐activating syndrome during therapy. As for late toxicities, 6 patients (6.9%) had thyroid disorders, and one patient developed metastatic papillary thyroid carcinoma 3 years after the end of therapy.

### 3.4 Treatment Response and Outcome

Remission was observed in 82 patients (94.2%) at the end of treatment. Refractory disease was observed in 5 patients (5.7%) while 9 patients (10.3%) had relapse. The outcome of the patients with rapid initial response after 2 OEPA showed long‐term remission in 18 out of 20 patients, while 2 patients relapsed. The median time to relapse for all patients was 8 months (IQR: 7–11) after the end of treatment. For refractory and/or relapse cases, several chemotherapy regimens were used, including IGEV (Ifosfamide, Gemcitabine, Etoposide, Vinorelbine, prednisolone), BvB (Brentuximab Vedotin, Bendamustin), ICE (Ifosfamide, Carboplatin, Etoposide), Pembrolizumab. Patients who achieved second remission received autologous transplant followed by RT, except for one patient who received RT alone without transplant and another patient received autologous transplant without RT.

### 3.5 Survival

**FIGURE 2:**
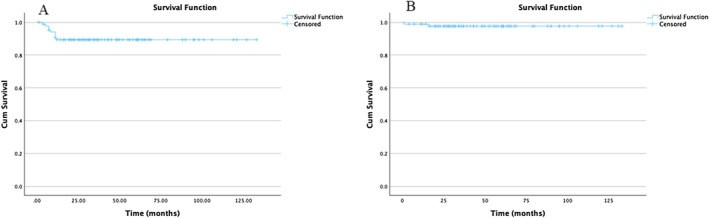
Kaplan–Meier survival curve for all groups. (A) Event‐free survival (EFS = 89.3%). (B) Overall survival (OS = 97.3%).

**FIGURE 3:**
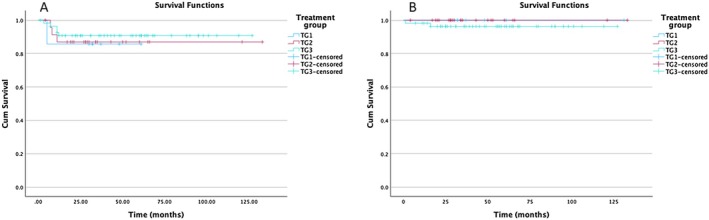
Kaplan–Meier survival curve stratified by treatment group. (A) Event‐Free Survival (EFS) (TG1 = 85.71%; TG2 = 86.96%; TG3 = 90.77%). (B) Overall survival (OS) (TG1 and TG2: 100.00%, TG3 = 96.3%).

Patients were followed up for five years after the end of treatment, with a mean follow‐up period of 49.9 ± 30.7 months. At the latest follow‐up, 85 patients (97.7%) were alive with complete remission. Death occurred in 2 patients: one patient died following macrophage‐activating syndrome secondary to EBV infection, and the other patient died from his disease despite multiple lines of treatments. The five‐year EFS and OS for all groups were 89.3% and 97.6%, respectively (Figure 2). The subgroup analyses showed EFS and OS rates of 85.7% and 100% for TG1, 86.9% and 100% for TG2, and 90.8% and 96.3% for TG3, respectively, at 5 years (Figure 3).

### 3.6 Prognostic Variables

Several prognostic variables were studied in order to investigate a potential association with survival and relapse. On univariate analysis, no association was found between relapse and the following parameters: age, gender, B‐symptoms, bulky disease, high ESR, staging, TG, family history of malignancy, extra nodal involvement, early response on PET‐CT, and administration of radiotherapy (Table 3). Furthermore, no independent factors were identified on multivariate analysis to significantly influence the EFS.

**TABLE 3:** Univariate Cox regressions on relapse‐free survival.

Variables	Crude HR (95% CI)	*p*
Age	1.09 (0.88, 1.34)	0.438
Age ≥ 13 years	1.90 (0.39, 9.14)	0.424
Female	0.73 (0.20, 2.73)	0.644
Positive family history of cancer	1.10 (0.30, 4.11)	0.884
Symptoms' duration (weeks)	0.98 (0.91, 1.04)	0.464
B‐symptoms	2.32 (0.48, 11.16)	0.294
ESR (mm)	1.00 (0.99, 1.02)	0.704
ESR ≥ 33 mm	1.61 (0.40, 6.43)	0.502
Lung	2.65 (0.71, 9.88)	0.146
Spleen	0.99 (0.25, 3.97)	0.991
Liver	0.05 (0.00, 221,991.08)	0.697
Bone	1.13 (0.23, 5.44)	0.879
Bone marrow	1.26 (0.16, 10.05)	0.830
Bulky disease	2.11 (0.57, 7.86)	0.266
Positive interim PET	0.63 (0.13, 3.01)	0.559
*Staging (Reference: I and II)*		
III	0.34 (0.04, 3.06)	0.337
IV	0.81 (0.20, 3.22)	0.760
*Treatment group (Reference: TG1)*		
TG2	0.85 (0.09, 8.16)	0.887
TG3	0.59 (0.07, 5.05)	0.630
Time to start chemotherapy (days)	0.89 (0.77, 1.03)	0.114
Radiotherapy	0.76 (0.19, 3.05)	0.701

Abbreviations: ESR, erythrocyte sedimentation rate; PET, positron emission tomography; TG, treatment group.

## 4 Discussion

The use of risk‐adapted chemotherapy with minimizing the need for RT has become the new standard of care to treat children with HL. While many western countries have implemented the Euronet‐HL protocol into their daily practice with excellent results, the application of this protocol in the LMIC settings is limited. This current study is a unique report from Lebanon and the MENA region.

Our cohort had a mean age of 13.1 years, with a similar frequency between males and females. These findings were comparable to those of Western countries [27]. Nevertheless, they contrast with previous results from our center during the period between 1980 and 1996 [28], as well as with studies from LMICs, where patients with HL tend to be younger (mean age around 8 years), and exhibit a male predominance [18, 19, 22, 29]. The high incidence of younger age groups affected by HL is thought to be correlated to early exposure to the EBV virus, particularly those from lower socio‐economic group [30] or LMICs [31]. These findings were confirmed in our previous study, where all patients under 13 years of age had positive EBV serology [28]. Although our current study showed a high exposure rate (58.6%) to EBV, the older population in our cohort could be explained by an intermediate epidemiological pattern of EBV infection in Lebanon, where EBV exposure happens at an older age [32]. Additionally, selection bias could have been a contributing factor, as adolescents in our settings are treated in pediatric facilities, unlike in some LMIC centers where patients over 12 years are treated by adult oncologists [19]. Moreover, the male predominance in younger patients has not been well understood, but it may be related to gender discrimination because girls tend to be neglected, as reported in some studies from LMICs [29, 33]. In Lebanon, both genders have equal access to medical attention.

B‐symptoms were among the first reported high‐risk features in HL [34]. In our cohort, 59% of our patients had B‐symptoms, which is similar to other reports from LMICs (46% in India [19], 55% in Pakistan [22]), but higher than European studies (37% of patients) [7, 8]. This could partially explain the elevated initial ESR in our cohort (57% of the patients) and the low incidence of patients treated in TG1 (8%).

The incidence of bulky disease in our cohort was 37%, which is similar to European studies (33%) [7, 8] but lower than LMICs (58%) [19, 22]. This discrepancy could be explained by differences in measurement techniques and/or the definition used for bulky disease. These latter studies defined “bulky disease” as a single conglomerate lymph node with a diameter of more than 6 cm or a mediastinal mass more than one‐third of the chest diameter. In contrast, the Euronet‐HL protocol requires the conglomerate lymph node volume to be more than 200 mL to be considered “bulky”. This divergence in definitions could have led to an overestimation of bulky disease in other studies.

Our findings demonstrated excellent results, with 94.2% of patients achieving complete remission, along with a high EFS of 89.3% and OS of 97.6% at 5 years. These results were similar to those of the Euronet‐PHL‐C1 trial as described by Landman‐Parker et al. [7, 8], and to other international studies [35] from high‐income countries (HIC). Reports from LMICs using the Euronet‐based protocol had slightly lower outcomes. For instance, Palayullakandi et al. from India [19] reported an EFS = 86.2% and OS = 93.5%, while Ghafoor [22] from Pakistan reported EFS and OS of 80.2% and 91.5%, respectively. Furthermore, these two studies were associated with increased treatment‐abandonment rates and high treatment‐related mortality, reflected by an elevated rate of FN episodes of 66 and 69 episodes in the Indian and Pakistani studies, respectively. In contrast, none of our patients abandoned therapy, and no toxic deaths were reported. This observation could be explained by the systemic use at our center of G‐CSF after each cycle of chemotherapy, which helped decrease the incidence of FN to 36 episodes. The addition of G‐CSF could improve supportive care, especially in LMIC settings.

The classic prognostic factors for HL‐B‐symptoms, bulky disease, and elevated ESR did not influence the survival or the relapse rate in our study. Furthermore, no associations or independent factors were found to correlate with the outcome. This implies that treatment intensification was adequately allocated to the category of patients with an expected high risk of treatment failure. As a result, we had a low relapse rate of 10.3%, which is comparable to reports from HIC [7, 8, 36]. However, the events occurred relatively early, around 8 months after the end of therapy, compared to approximately 2 years, according to international reports [36]. Recent studies have attributed early relapse/refractory disease in HL to incomplete eradication of cancer cells due to a disruptive interaction between inflammatory cells and the immune system in the tumor microenvironment [37, 38, 39]. The majority of the relapsed cases in our study had B‐symptoms and elevated ESR, suggesting a high inflammatory burden. Furthermore, these patients had already received consolidative treatment with RT since they were slow responders, indicating that we were dealing with highly aggressive disease. Fortunately, we were able to salvage these patients by using second‐line chemotherapy followed by stem cell transplantation, which yielded a good response and increased the OS to 97% at 5 years.

One of the primary objectives of the Euronet‐HL protocol was to omit RT for rapid responders to prevent long‐term toxicities since the speed of response was found to have a significant prognostic value in previous studies. Our data revealed that RT was omitted in only 22.9% of patients (42.8% in TG1, 45.8% in TG2, and 10.7% in TG3), which is lower than in the Euronet study, where RT was omitted in approximately 47% of patients (68% in TG1 [7] and 40% in TG2 and TG3 [8]). Our results also differ from those of the Indian study [19], where RT was spared in 65.6% of patients based solely on PET‐CT results, with an additional 17 patients not receiving RT due to physician discretion. On the other hand, the Pakistani study [22] reported that 77.7% of patients exhibited an early response, primarily assessed by CT scans, as PET‐CT was not widely available; consequently, these patients did not receive RT. The difference in how early response is evaluated in both studies may explain the discrepancy in the number of patients receiving RT. An analysis of our “rapid responders” group revealed that 55% of patients were assigned to TG2, and the only 2 patients who relapsed had bulky disease, with relapse sites involving both bulky and distant locations. In contrast, all patients in the Indian study who did not receive RT relapsed. Additionally, no outcomes were reported on the Pakistani patients for whom RT was spared. In the early days of implementation of Euronet in our center, physicians were hesitant about omitting RT for good responders. One reason was that one of the earliest “rapid‐responder” patients in 2012, assigned to the TG1 group, relapsed 5 months after completing therapy, but later successfully salvaged with radiotherapy alone. Another contributing factor was the results of the interim analysis of the Euronet study, which reported an increased relapse rate in the TG1 group. Consequently, a total of 17 patients received RT despite being rapid responders and were excluded from the study analysis. Of these, 12 patients were in TG1 and had bulky disease, 2 were in TG2, and 3 were in TG3. Six patients had elevated ESR, and none of the 17 patients experienced relapse. Fortunately, after the Euronet group amended the protocol to upstage TG1 patients with bulky disease and elevated ESR to TG2, treating physicians became more confident in omitting RT for rapid responders, leading to more patients being spared RT.

Although bulky disease was not identified as a prognostic factor for relapse in our cohort, this may be due to the small sample size and retrospective nature of our study. In the broader literature, bulky disease remains a recognized adverse prognostic factor often warranting radiotherapy [7, 8, 40, 41, 42]. In an effort to enhance survival while reducing reliance on RT, several trials have explored incorporating novel agents into first‐line therapy. In a non‐randomized study, Metzger et al. were among the first to combine brentuximab vedotin with chemotherapy [43]. They demonstrated that RT could be safely omitted in a substantial proportion of high‐risk patients, including those with bulky disease, while achieving excellent OS. However, 65% of children still received RT. Moreover, the randomized trial by Castellino et al. showed similar excellent survival when substituting bleomycin with brentuximab vedotin but failed to reduce the use of RT [40, 44]. Nonetheless, these outcomes were achieved in high‐income settings with access to comprehensive salvage therapies. In LMICs settings like Lebanon, where resource limitations and economic constraints impair the feasibility of intensive salvage regimens, the use of these agents is often too costly to be incorporated into first‐line therapy. Therefore, the omission of RT may carry a considerable risk of relapse, thereby supporting its continued use in high‐risk patients or bulky disease. Consequently, we opted to optimize first‐line therapy with RT for slow responder patients with strict definitions (residual disease on CT > 25% and Deauville Score > 3 on PET‐CT) and not to follow the new Euronet‐PHL‐C2 criteria (residual disease on CT > 50% and Deauville Score > 4 on PET‐CT).

One could argue that reinforcing treatment with RT could increase toxicity and secondary neoplasm. However, our findings showed that the majority of the children tolerated the therapy well, with manageable toxicities and a low rate of secondary neoplasm. To further mitigate the long‐term side effects of RT, one suggestion is to administer RT only to residual lymph nodes with persistent FDG activity on PET‐CT after 2 OEPA, rather than to the entire involved field, after the encouraging results of Ozuah et al. [45]. However, the impact of these measures on our patients remains to be evaluated in long‐term randomized studies.

This study is among a few reports that have evaluated the outcome of Hodgkin Lymphoma in children from the MENA region using the Euronet‐HL protocol. We have demonstrated the successful implementation of a risk‐adapted, response‐based strategy without reliance on costly medications, achieving high survival rates and reducing the use of RT through careful selection of low‐risk, good‐responder patients. Nevertheless, our study had some limitations, including its retrospective design, a single‐center experience, and a small sample size. Additionally, the 5‐year follow‐up for our patients may not be sufficient to detect potential long‐term toxicities, particularly secondary neoplasms, which often take a longer time to develop.

## 5 Conclusion

The shift toward risk‐adaptive chemotherapy and minimizing reliance on radiotherapy marks a significant advancement in pediatric Hodgkin lymphoma treatment; however, its implementation in LMICs remains limited. This study provides a unique experience from Lebanon, where the adoption of the Euronet‐HL protocol provided excellent outcomes and a safety profile with a prolonged OS rate reaching 97.6% at 5 years despite several challenges. The incorporation of G‐CSF into chemotherapy has markedly reduced the rate of FN and infections. Based on this experience, fewer patients with low‐stage, rapid‐responder HL may require irradiation. However, oncologists in resource‐limited settings remain cautious about removing RT for patients with advanced stage or with bulky disease. Prospective trials in LMIC settings are needed to establish clearer guidelines for identifying patients for whom RT can be safely omitted.

## Author Contributions

Conceptualization: Elie Bechara, Samar Muwakkit. Methodology: Elie Bechara, Arwa El‐Dhuwaib. Data curation: Arwa El‐Dhuwaib, Hani Tamim. Investigation: Elie Bechara, Toufic Eid, Arwa El‐Dhuwaib. Validation: Elie Bechara, Toufic Eid, Hani Tamim, Dolly Noun, Raphah Borghol, Zaher Chakhachiro, Miguel R. Abboud, Samar Muwakkit. Formal analysis: Elie Bechara, Hani Tamim, Samar Muwakkit. Supervision: Samar Muwakkit. Writing – original draft: Elie Bechara. Writing – review and editing: Elie Bechara, Toufic Eid, Arwa El‐Dhuwaib, Hani Tamim, Dolly Noun, Raphah Borghol, Zaher Chakhachiro, Miguel R. Abboud, Samar Muwakkit.

## Conflicts of Interest

The authors declare no conflicts of interest.

## Data Availability

The data that support the findings of this study are available from the corresponding author upon reasonable request.
